# The Hop Polyphenols Xanthohumol and 8-Prenyl-Naringenin Antagonize the Estrogenic Effects of *Fusarium* Mycotoxins in Human Endometrial Cancer Cells

**DOI:** 10.3389/fnut.2018.00085

**Published:** 2018-09-19

**Authors:** Georg Aichinger, Julia Beisl, Doris Marko

**Affiliations:** Department of Food Chemistry and Toxicology, University of Vienna, Vienna, Austria

**Keywords:** flavonoid, xenoestrogen, mycoestrogen, interaction, combination index, synergism, endocrine disruption

## Abstract

The *Fusarium* toxin zearalenone (ZEN) and its reductive metabolite α-zearalenol (α-ZEL) are well-documented endocrine disruptors that are frequently found to contaminate cereal products, including beer. But also hop is known to represent a source for endocrine active compounds, containing amongst others xanthohumol (XAN), which might be converted to the potent phytoestrogen 8-prenylnaringenin (8-PN). In the present study, we investigated the interaction of these xenoestrogens in mixtures which might occur in beer. Estrogenicity was measured as induction of alkaline phosphatase (AlP) expression in estrogen-sensitive Ishikawa cells. In binary combinations, XAN was found to act as a potent antagonist of mycotoxin-induced estrogenicity, significantly suppressing the AlP-inducing impact of both ZEN and α-ZEL at nanomolar concentrations. Also 8-PN antagonized the estrogenic stimulus of the two fungal metabolites, although less pronounced. These effects also manifested in combinations of three or four test compounds, and at the level of cell proliferation, that was assessed via an E-screen-like approach in Ishikawa cells. Of note, co-exposure to the investigated myco- and phyto-estrogens did not result in additive or overadditive/synergistic estrogenic effects in the applied test system. Being aware that the actual study is still limited to the *in vitro* situation, our results even suggest that prenylated chalkones from hops might protect against *Fusarium* toxin–induced endocrine disruptive activities at concentrations that can be reached by moderate beer consumption.

## Introduction

**Figure 1:**
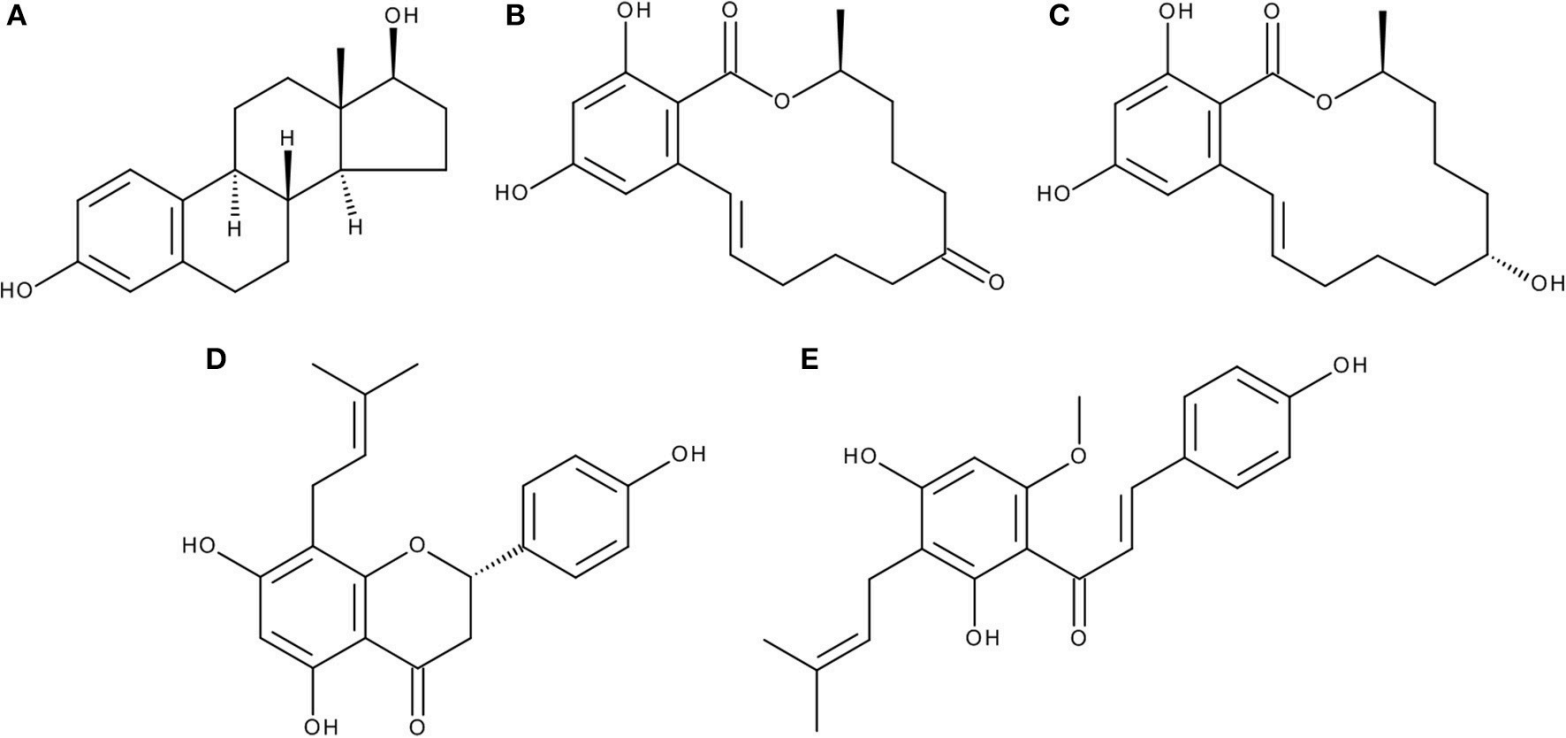
Chemical structures of **(A)** 17β-estradiol (E2), **(B)** zearalenone (ZEN), **(C)** α-zearalenol (α-ZEL), **(D)** 8-prenylnaringenin (8PN), and **(E)** xanthohumol (XAN).

Mycotoxins, toxic secondary metabolites of molds, are probably the most frequently found food contaminants and pose a potential threat to food safety and thus to human health. Among those, some mycotoxins have been reported to structurally mimic the natural hormone 17β-estradiol (E2, Figure 1A) and thus to bind to and activate estrogen receptors (ER), which could potentially lead to adverse effects like enhanced proliferation of estrogen-sensitive tissues, problems with sexual development, impairment of fertility or other symptoms of a disturbed hormone balance, the so-called “endocrine disruption.”

The probably most “famous” mycotoxin which acts in that way is zearalenone (ZEN, Figure 1B), a full agonist for ER-α(1), which has been reported to cause a variety of adverse effects in animals and humans (2). ZEN is a secondary metabolite produced by *Fusarium* molds, which are often found to infest corn or other grains (3). Consequently, ZEN and other *Fusarium* toxins are found in animal feed (4), cereals, bakery products, or beer (5).

While ZEN is thermally stable and thus remains in food after cooking, it is metabolized by both the gut microbiota and the human liver, resulting in different metabolites including, among others, α-zearalenol (α-ZEL, Figure 1C) and β-zearalenol. Of these, especially α-ZEL is of high relevance, due to its higher estrogenic potency as compared to ZEN and its occurrence in relevant concentrations after ingestion of ZEN.

The endocrine impact of the latter compounds is extensively described in scientific literature (6), but considerably less attention has been attributed to the question on the combinatory impact of these mycoestrogens in mixture with other co-occurring xenoestrogens. The analysis of combinatory effects of chemical mixtures is of growing interest in the field of toxicology and risk assessment (7). Recently, we have shown another estrogenic mycotoxin, alternariol, to potentiate estrogenic effects of *Fusarium* toxins *in vitro* (8). But also the soy isoflavone genistein, a well-described phytoestrogen, was found to interact synergistically with zearalenone in an ER-dependent reporter gene assay (9). Of consequence, it seems to be of fundamental importance for risk assessment to investigate further potential interactions between xenoestrogens in food.

One of the most potent phytoestrogens known today is 8-prenylnaringenin (8-PN, Figure 1D), a polyphenolic prenylated chalcone found in hops (*Humulus lupulus*), which has been described as a ligand for both ER-α and ER-β, acting as a full agonist (10). The prime source of human exposure is beer, with concentrations usually around 100 μg/l (11). But 8-PN is also contained in herbal formulas for treating menopause-related conditions and for breast enhancing purposes (10), which should be considered skeptically, as the compound was also shown to enhance mammary gland tissue growth and thus is suspected to promote related tumor growth (12). However, even as the use as a herbal medicine might have a problematic impact, the compound is not expected to exert adverse effects at the concentrations available in beer (13). 8-PN can be formed by both the hops plant and the microbiota of the human gut via the demethylation of iso-xanthohumol (iso-XAN), which in turn results from the isomerization from xanthohumol (XAN, Figure 1E), the predominant flavonoid found in hops (14). Unlike 8-PN, the parent compound XAN does not act as an activator of ERs. On the contrary, there are reports of XAN being able to act in an anti-estrogenic way (15). XAN has also been described as an anti-oxidant agent, leading brewers to produce XAN-enriched beers to promote supposed positive health effects of their beer, which consequently might lead to a higher overall consumption of prenylated chalcones (16, 17).

Of note, humans might be simultaneously exposed to both *Fusarium* mycotoxins and hop polyphenols, either by co-occurrence in food (e.g., in beer brewed from contaminated grains) or by a simultaneous ingestion of beer and contaminated food. Regarding interactions of polyphenolic flavonoids with mycotoxins, there are quite a few studies describing protective effects of the former against cytotoxic or genotoxic effects of mycotoxins (18–21). However, we recently described the soy isoflavone genistein to potentiate estrogenic effects of ZEN in a human cancer cell line, leading to an induction of ER-related gene expression that even exceeded the levels induced by E2, the natural ligand (9). Thus, it seems possible that prenylated chalcones might interact in a similar way. On the other hand, it might also be speculated about protective effects, since XAN was previously reported as an anti-estrogenic compound.

To address these pending questions, we expanded our research on the beer polyphenols 8-PN and XAN, and we combined those two with the Fusarium metabolites ZEN and α-ZEL to search for interactions regarding their estrogenic impact in the Ishikawa cell model.

## Materials and methods

### Chemicals and enzymes

XAN was purchased from Extrasynthese (Genay, France). 8PN, ZEN, and α-ZEL were obtained from Sigma-Aldrich (Taufkirchen, Germany). Media and supplements for cell culture were purchased from Invitrogen^TM^ Life Technologies (Karlsruhe, Germany). Live Cell Imaging Solution (LCIS), Hoechst 33342, and CellMask^TM^ Deep Red Plasma Membrane Stain were obtained from Thermo Fisher Scientific.

### Cell culture

The Ishikawa human endometrial cancer cell line was obtained from ECACC (Wiltshire, UK). Stocks were stored in liquid nitrogen. Cells were grown in Minimum Essential Medium (MEM), supplemented with 5% (*v/v*) heat inactivated fetal calf serum (FCS), 1% l-glutamine and 1% (*v/v*) penicillin/streptomycin, under humified conditions, with 5% CO_2_, at 37°C. Monitoring of potential mycoplasma contaminations was routinely performed. For experiments, cells were seeded in assay medium (DMEM/F12 medium containing 5% charcoal-dextrane stripped FCS (CD-FCS) and 1% (*v/v*) penicillin/streptomycin). The same medium was used for incubations with the test compounds that were dissolved in dimethyl sulfoxide (DMSO), resulting in a final concentration of 0.1–0.2% DMSO in the incubation solutions, depending on the experiments.

### Alkaline phosphatase (ALP) assay

The AlP assay was based on the method by Littlefield et al. (22), and optimized as previously described (8). Briefly, 10,000 Ishikawa cells/well were seeded in 96-well plates and allowed to grow for 48 h using assay medium. Then they were incubated with the test compounds, compound combinations or the solvent control in assay medium for another 48 h in triplicates. Afterwards, incubation solutions were removed and the cells were washed three times with phosphate buffered saline (PBS). The wells were sucked dry and the plates were frozen at −80°C for 20 min. Afterwards, they were allowed to thaw at room temperature for 5 min, and 50 μl AlP buffer (1 M diethanolamine, 5 mM 4-nitrophenylphosphate, 0.24 mM MgCl2, pH 9.8) were added into the wells. The increase of absorption at 405 nm was monitored with a plate reader every 3 min for 1 h, and the slope of the linear range of the resulting curve was used for calculating the induction of AlP expression in relation to the respective solvent control [0.1–0.2% (*v/v*) DMSO].

### Cell proliferation assay

An induction of the ER pathway is commonly linked with increased proliferation of estrogen-sensitive cells. Thus, a standard assay for assessing the estrogenic potency of a compound is the so-called “E-screen assay” that uses the MCF-7 cell line and was established by Soto et al. (23). We adapted this assay for the use of Ishikawa cells. 600 cells/well were seeded in 96-well plates and allowed to attach for 24 h. Afterwards, they were incubated with the test compounds diluted in 100 μl of DMEM/F-12 medium, containing 5% CD-FCS, 1% penicillin/streptomycin and a final solvent concentration of 0.15% (*v/v*) DMSO in triplicates. Incubation solutions were renewed after 3 days to ensure the sufficiency of nutrients in the media. After a total of 6 days of incubation, cells were fixed by addition of 10 μl/well of a trichloroacetic acid solution (50% *m/v*). After 1 h of storing at 4°C, the plates were washed four times with water and were allowed to dry overnight. Subsequently, wells were incubated with a 0.4% (*w/v*) solution of sulforhodamine B (SRB) at room temperature for 1 h. After four times washing with 1% *(v/v)* acetic acid, wells were allowed to dry overnight. Finally, 100 μl/well of Tris buffer (pH 10) was added to resolve protein-bound SRB and the absorbance of the wells' content was measured at 570 nm with a PerkinElmer Victor^3^V plate reader. All measured values were related to the respective solvent control [0.1–0.2% (*v/v*) DMSO].

### Cell imaging

After 24 h of growth, cells were incubated with a representative concentration of each XAN, ZEN, and α-ZEL, both as a single compound and in combination as described for the cell proliferation assay. 0.2% *(v/v)* DMSO served as a solvent control, and 1 nM E2 as the positive control. At day 0, 3, and 6, bright field images of the cells were taken with a Biotek Cytation 3 plate reader equipped with a 20x objective. In addition, at day 3 and 6, one well of each sample incubation was stained with Hoechst 33342 solution (10 μg/ml in LCIS) and CellMask^TM^ Deep Red Plasma Membrane Stain (1:1000 in LCIS) for 30 min, washed twice with PBS and refilled with 100 μl LCIS. Subsequently, fluorescence images was captured with the equipment described above (λ_ex_ = 350 nm, λ_em_ = 461 nm).

### Statistical analysis—the combination index

An interaction is defined as the combinatory effect of two or more compounds being “not additive,” but rather synergistic (“more than additive”) or antagonistic (“less than additive”). To assess interactions, mathematical models are required, as the intuitive formula:

$$\begin{array}{l} \;additive\;effect=effect\;1+effect\;\text{2} \end{array}$$

only applies to linear dose-response relationships, which hardly ever occur in biological systems. The most advanced statistical model today is the combination index (CI) theorem of Chou and Talalay (24), that is universally valid regardless of curve shape or type of interaction and provides a calculated factor as a measure for the type and strength of an interaction. In general, a CI > 1 is considered antagonistic, while a CI < 1 is describing a synergism. However, we adopted the apportionment of how to describe the strength of an interaction (Table 1), which Chou introduced in the latest amendment to his model (25). We designed our AlP assays to meet the two requisitions of the CI model: a concentration range where each compound would reach an effect of 50% and a constant concentration ratio of the applied compounds. Thus, the CI theorem was used for interaction analysis in all AlP assays, with exceptions for the binary combinations including XAN, as this compound was not estrogenic in our test system and thus an ED_50_ value could not be obtained. Here, we just compared the measured combinatory effect to the effect of the single estrogenic compound using Student's *t*-test. For our situation, the key takeaways of the CI model were the CI_50_ value (i.e., the CI at 50% effect), its graphical display (the “isobologram”) and the effect-CI plot, that describes the development of the synergistic/antagonistic strength over the whole effect range.

**Table 1:** Relationship between CI values and the potency of an interaction.

**CI**	**Description of interaction**
<0.1	Very strong synergism
0.1–0.3	Strong synergism
0.3–0.7	Synergism
0.7–0.85	Moderate synergism
0.85–0.9	Slight synergism
0.9–1.1	Nearly additive
1.1–1.2	Slight antagonism
1.2–1.45	Moderate antagonism
1.45–3.3	Antagonism
3.3–10	Strong antagonism
>10	Very strong antagonism

*Classification according to (25)*.

## Results

### ALP assay: single compounds

**Figure 2:**
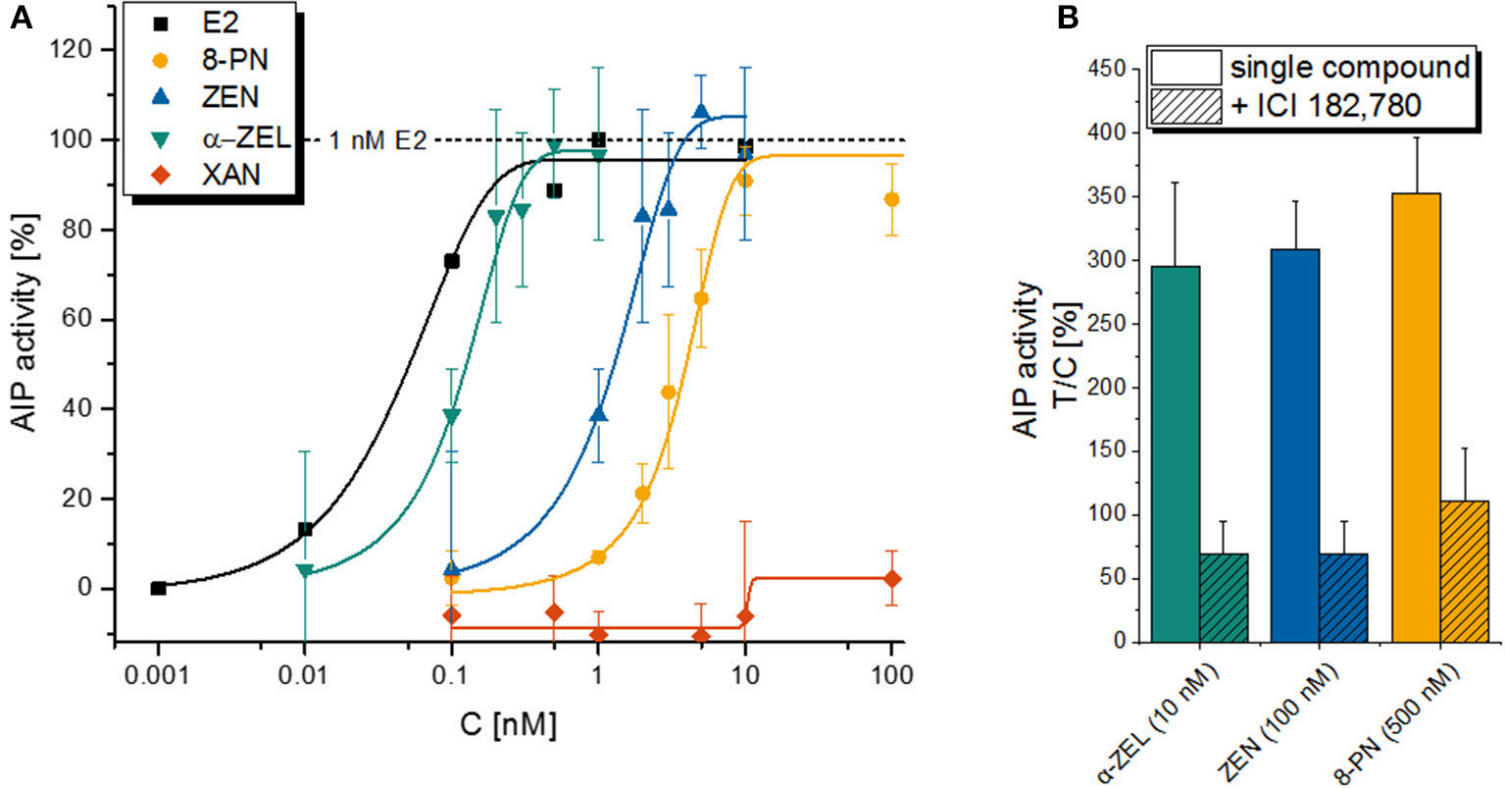
Alkaline phosphatase (AlP) assay with single compounds. Graph **(A)** is depicting the concentration-dependent increase in AlP activity as a measure of estrogenic activity for a 48 h incubation with E2, α-ZEL, ZEN, 8PN or XAN in Ishikawa cells. Data points are expressed as means ± SD of at least 3 independent experiments (except for E2, where a single experiment is shown) in relation to the positive control (1 nM E2). Graph **(B)** is showing the suppression of the latter effect by the selective ER antagonist ICI 182, 780 for the 3 active xenoestrogens. Again, values are expressed as mean + SD for at least 3 independent experiments related to the solvent control [0.1% *(v/v)* DMSO].

As expected, an incubation with the estrogenic test compounds lead to a concentration-dependent increase of AlP activity in Ishikawa cells, with the natural estrogen E2 as the strongest inducer with an ED_50_ of 0.051 nM, followed by α-ZEL (0.12 nM), ZEN (1.3 nM), and 8-PN (3.9 nM) as calculated by a non-linear curve fit (Figure 2A). XAN did not show any estrogenic effect as a single compound. The induction of AlP expression was confirmed to be a consequence of ER activation for all compounds, as the co-incubation with the selective ER inhibitor ICI 182.780 (ICI) suppressed AlP activity (Figure 2B).

From this preliminary results, combination ratios were chosen that would reflect a seemingly realistic situation and ensure that all involved compounds (except the inactive XAN) would be active in the chosen concentration range.

### ALP assay: binary combinations

**Figure 3:**
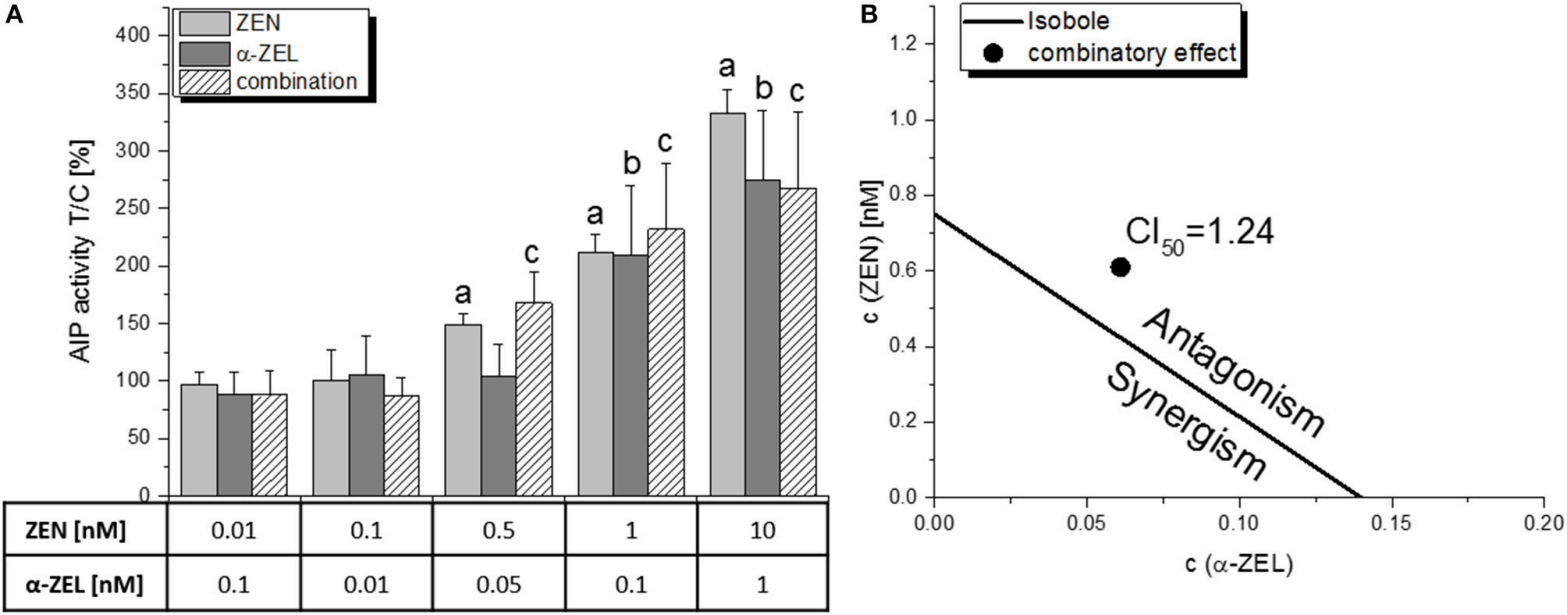
The combinatory estrogenic effect of ZEN and α-ZEL, as measured by the AlP assay. **(A)** shows the results of the single compounds and the combination of at least 5 independent experiments, expressed as means + SD and related to the solvent control [0.1% *(v/v)* DMSO]. Significant differences to the respective no-effect dose were calculated by one-way ANOVA (*p* < 0.05), followed by Fisher's LSD *post-hoc* testing, and are indicated with “a” (ZEN), “b” (α-ZEL) or “c” (combination). **(B)** shows the isobologram as a graphical display of the CI_50_ with a line (the “isobole”) connecting ED_50_ values of ZEN and α-ZEL, and the ED_50_ value of the combination lying on the right side of the isobole, indicating an antagonistic interaction.

The combined effect of ZEN and α-ZEL (ratio 10:1, Figure 3A) was – however insignificantly - higher than the effects of the single compounds at 0.05 nM and 0.1 nM α-ZEL (0.5 nM and 1 nM ZEN, respectively), but was found to be “moderately antagonistic” at the ED_50_ level with a CI_50_ of 1.24, which can be visualized in an isobologram (Figure 3B). This graphical display of the CI calculation indicates that the concentrations to reach the ED_50_ for the combination of the two compounds were slightly higher than to be expected from the dose-response curves of the single compounds, indicating a limited antagonistic interaction.

**Figure 4:**
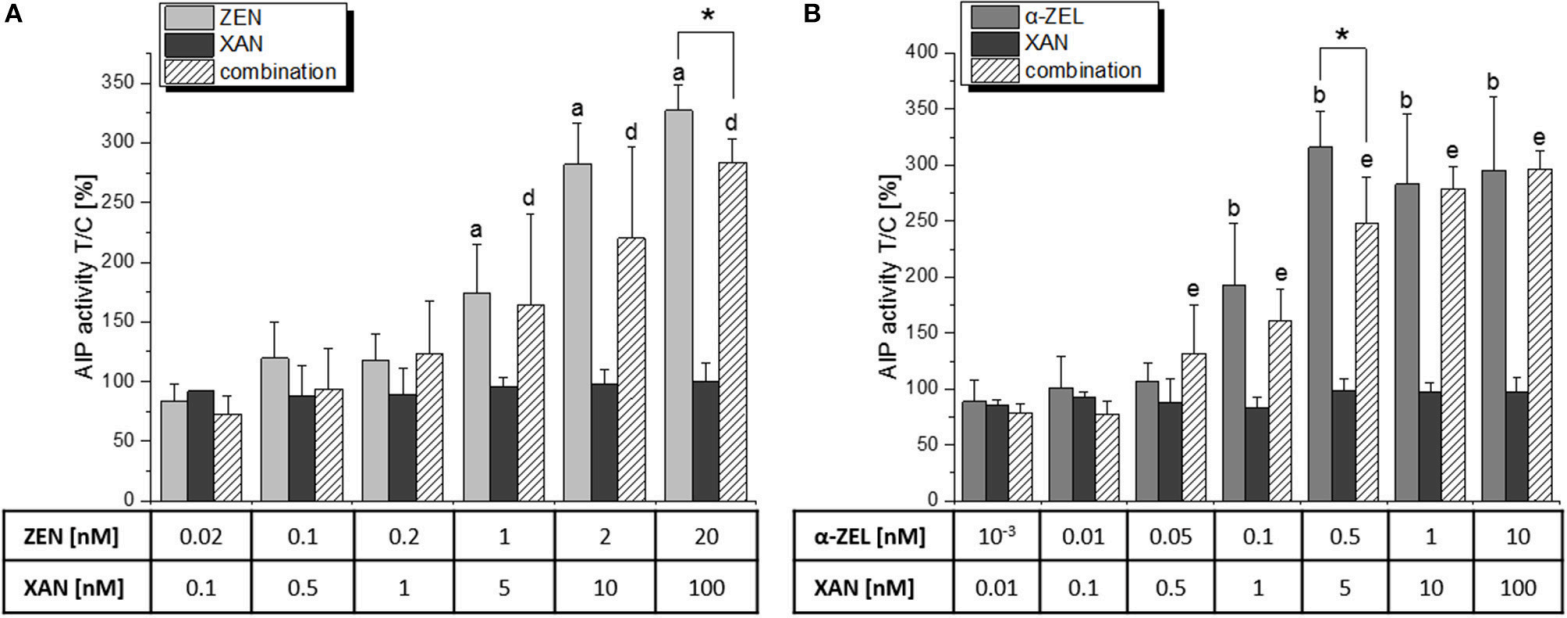
AlP assay results of a combination of XAN with **(A)** ZEN and **(B)** α-ZEL, expressed as means + SD of at least 5 independent experiments in relation to the solvent control [0.1% *(v/v)* DMSO]. Significant differences to the respective no-effect dose were calculated by one-way ANOVA (*p* < 0.05), followed by Fisher's LSD *post-hoc* testing, and are indicated with “a” (ZEN), “b” (α-ZEL), “c” (XAN), “d” (XAN/ZEN), or “e” (XAN/α-ZEL). Significances between effects of a single compound and the respective combination were calculated by Student's *t*-test and are indicated with “*” (*p* < 0.05).

For the combination of XAN with each of the two mycotoxins (5:1 for ZEN, 10:1 for α-ZEL), the application of CI calculations was not possible as XAN did not act estrogenic itself. However, the presence of XAN seemed to reduce the estrogenic impact of both compounds (Figure 4). More specifically, XAN significantly impaired the induction of AlP by the mycotoxins at the concentrations where their upper plateau of activity was reached (20 nM ZEN or 0.5 nM α-ZEL). Thus, XAN exerted significant antagonistic interactions with both ZEN and α-ZEL.

**Figure 5:**
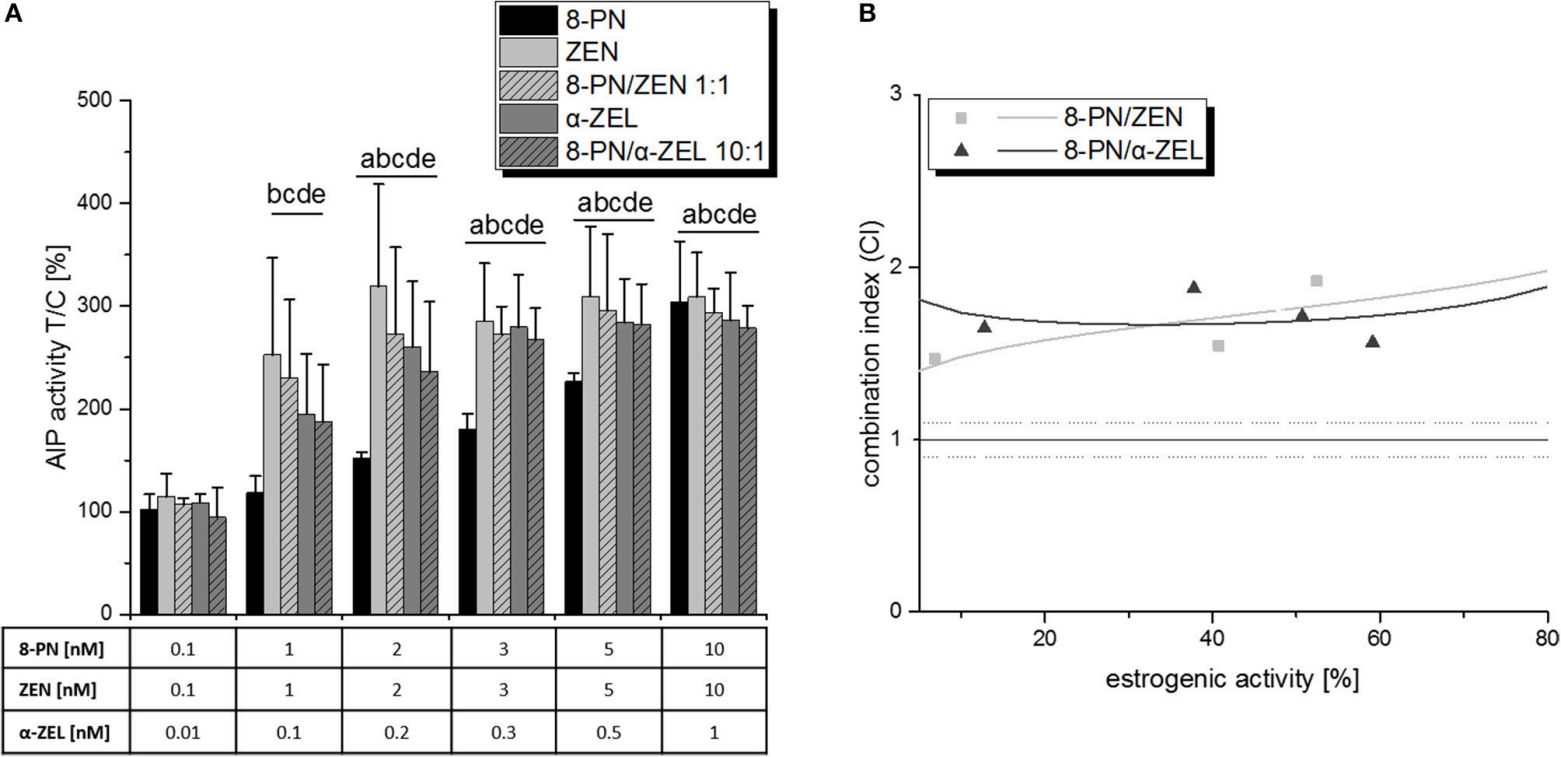
The influence of 8-PN on the estrogenic effects of ZEN and α-ZEL, as observed in the AlP assay. **(A)** shows AlP activities after 48 h of incubation, expressed as mean+SD of at least 5 independent experiments, in relation to the solvent control [0.1% *(v/v)* DMSO]. Significant differences to the respective no-effect dose were calculated by one-way ANOVA (*p* < 0.05), followed by Fisher's LSD *post-hoc* testing, and are indicated with “a” (8-PN), “b” (ZEN), “c” (8-PN/ZEN), “d” (α-ZEL), or “e” (8-PN/α-ZEL). The effect-CI plot for both combinations is shown in **(B)** with muttons and triangles indicating CI values at the measured concentration points and lines indicating the calculated behavior of the CI over the effect range.

Moreover, also the combination of the *Fusarium* metabolites with 8-PN lead to antagonistic interactions (Figure 5). Albeit no significant reduction of estrogenic activity was observed by comparison of single compound effects with respective combinatory effects, the combination index analysis revealed CI_50_ values of 1.76 for ZEN/8-PN (1:1) and 1.68 for α-ZEL/8-PN (1:10), both “antagonistic” according to Chou, as listed in Table 1). The corresponding effect-CI plots show an almost constant antagonistic interaction over the whole effect range (Figure 5B).

### ALP assay: combinations of higher order

**Figure 6:**
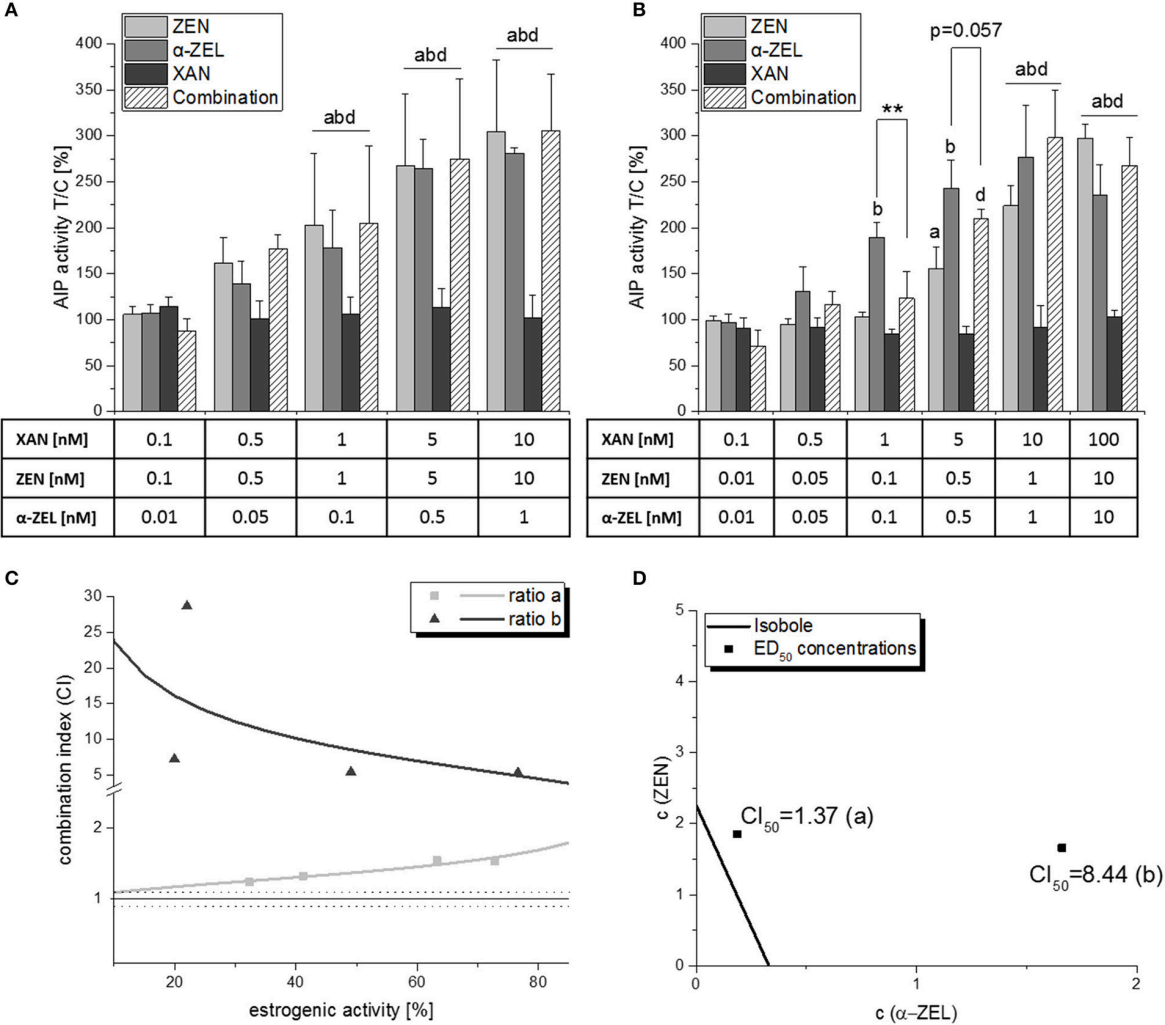
AlP assay results of ternary combinations of ZEN, α-ZEL and XAN in two different ratios (ratio a: ZEN:α-ZEL:XAN = 10:1:10; ratio b: ZEN:α-ZEL:XAN = 1:1:10). **(A,B)** show AlP activities after 48 h of incubation, expressed as mean ± SD of at least 3 independent experiments, in relation to the solvent control [0.1% *(v/v)* DMSO]. Significant differences to the respective no-effect dose were calculated by one-way ANOVA (*p* < 0.05), followed by Fisher's LSD *post-hoc* testing, and are indicated with “a” (ZEN), “b” (α-ZEL), “c” (XAN), or “d” (combinations). Significant differences between effects of a single compound and the respective combination were calculated by Student's *t*-test and are indicated with “**” (*p* < 0.01). Graph **(C)** shows the effect-CI plot of ratios **(A)** and **(B)**, and graph **(D)** the isobologram for these combinations.

Due to the significant reduction of mycotoxin-induced estrogenicity in binary combinations, we chose to combine XAN with both ZEN and α-ZEL simultaneously. In a concentration ratio of ZEN:α-ZEL:XAN = 10:1:10 (“ratio a”), a “moderately antagonistic” interaction was observed as indicated by the CI_50_ of 1.37 (Figure 6A). Surprisingly, the antagonistic interaction was much more pronounced with a reduced amount of ZEN, i.e., a higher amount of XAN per cumulative mycotoxin content (“ratio b,” ZEN:α-ZEL:XAN = 1:1:10, Figure 6B). Here, XAN significantly reduced the estrogenic impact of the combined mycotoxins at a concentration of 1 nM. This trend was also visible at 5 nM XAN, even if it was just not significant (*p* = 0.057). Furthermore, the CI calculation revealed a “strong antagonism” with a CI_50_ of 8.44 (Figure 6D).

Those differences are also clearly visible in the effect-CI plot, which revealed an antagonistic interaction over the whole effect range for both ratios, but substantially higher calculated CI values for ratio b (Figure 6C). We also visualized the ratio—dependent change in interaction strength in an isobologram (Figure 6D).

**Figure 7:**
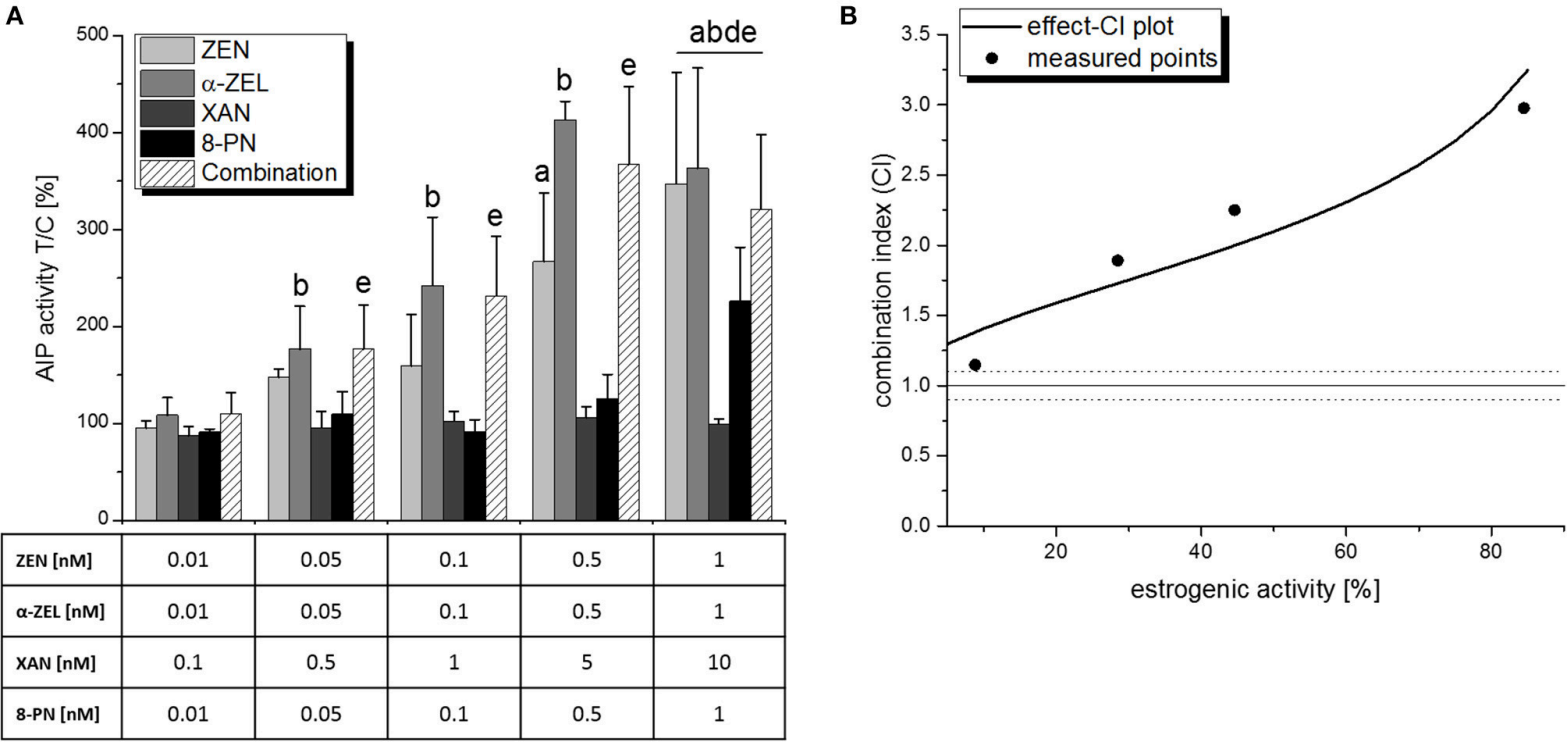
Combinatory estrogenic effects of all test compounds combined, as measured by AlP assays. **(A)** shows AlP activities after 48 h of incubation, expressed as mean+SD of at least 4 independent experiments, in relation to the solvent control [0.1% *(v/v)* DMSO]. Significant differences to the respective no-effect dose were calculated by one-way ANOVA (*p* < 0.05), followed by Fisher's LSD *post-hoc* testing, and are indicated with “a” (ZEN), “b” (α-ZEL), “c” (XAN), “d” (8-PN), or “e” (8-PN/α-ZEL). **(B)** shows the effect-CI plot with circles indicating CI values at the measured concentration points and lines indicating the calculated behavior of the CI over the effect range. The dotted line at *CI* = 1.1 represents the defined border of a “near additive” behavior.

After adding the phytoestrogen 8-PN to ratio b and thus creating a quaternary mixture, the strong antagonistic effect was less pronounced (Figure 7A). No significant reduction of AlP was observed in the measured combinations. However, combinatory effects did not exceed the effects of the most potent single compound (α-ZEL), and the combination index analysis consequently revealed an antagonistic behavior of the mixture over the whole effect range and an “antagonistic” CI_50_ of 2.1 (Figure 7B).

### Cell proliferation and morphology

**Figure 8:**
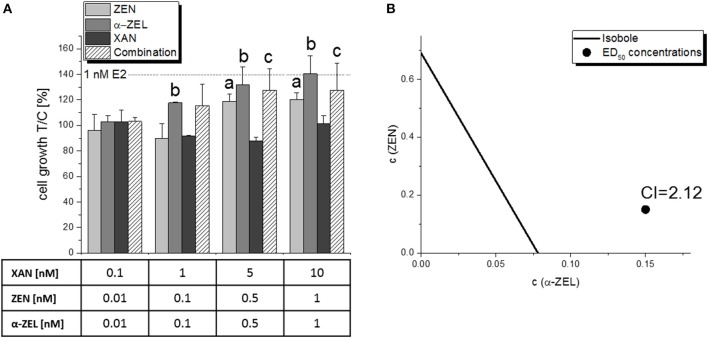
Impact on the growth of Ishikawa cells in the sulforhodamine B assay by a combination of XAN, ZEN, and α-ZEL (10:1:1, “ratio b”) after 2x 72 h of incubation. Columns of graph **(A)** show the measured protein content in relation to the solvent control [0.15% *(v/v)* DMSO] as means + SD of 5 independent experiments. Significant differences to the respective no-effect dose were calculated by one-way ANOVA (*p* < 0.05), followed by Fisher's LSD *post-hoc* testing, and are indicated with “a” (ZEN), “b” (α-ZEL), or “c” (combination). The effect caused by 1 nM E2, which was used as a positive control, is indicated with a dotted line. Graph **(B)** shows the isobologram produced from those results, with the CI indicating an antagonistic interaction.

The potential impact on cell proliferation was measured for the single compounds and the most relevant combinations of the AlP assays. Cell proliferation was determined in Ishikawa cells using an E-screen-like assay setup with a total incubation time of 2x 3 d, and a subsequent measurement of the total amount of cellular protein in each well using the SRB assay. All tested estrogenic compounds (E2, ZEN, α-ZEL, 8-PN) were found to induce cell growth in this assay (Figure S1). However, it was much less sensitive than the AlP assay and revealed mostly insignificant trends, with the exception of the combination of XAN:ZEN:α-ZEL in mixture “b” (10:1:1), which also showed also the strongest antagonistic interaction in the AlP assay (Figure 8A). For the impact on cell proliferation, a CI_50_ value of 2.12 was calculated, which indicates an “antagonism” (Table 1), and can be visualized in an isobologram (Figure 8B).

**Figure 9:**
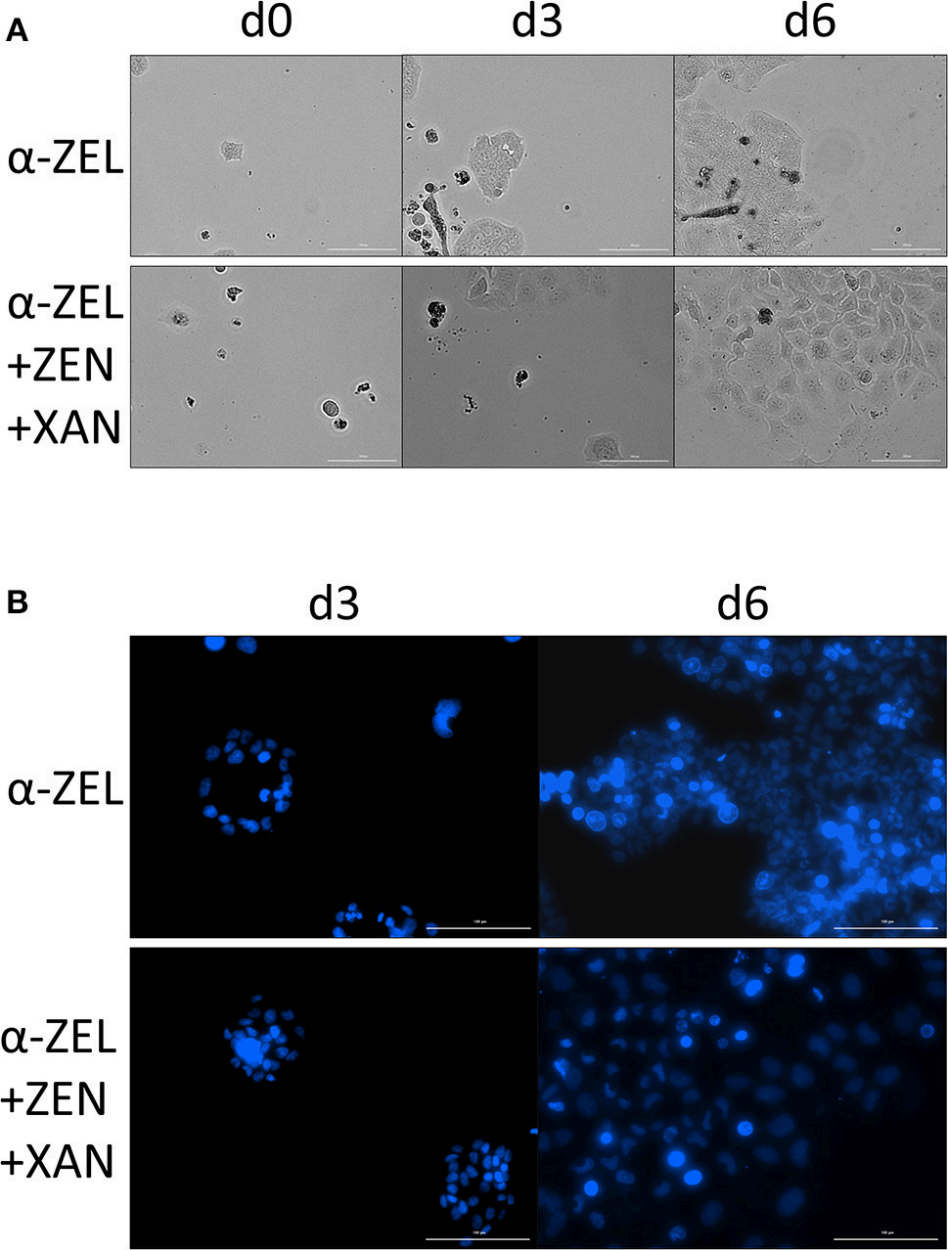
Changes in the morphology of Ishikawa cells. Images show a comparison of an incubation with 1 nM α-ZEL to the respective combination of 1 nM α-ZEL, 1 nM ZEN and 10 nM XAN. **(A)** shows bright field images after an incubation time of 0, 3, and 6 d, with the combination to yield larger cells in general. **(B)** shows nuclei stained with Hoechst 33242 after 3 and 6 d, with the combination resulting in larger and less dense nuclei. The white line in each of the images indicates a distance of 100 μm.

After the same type of incubations, images of the cells were taken both in bright field mode and with Hoechst staining. As compared to α-ZEL as a single compound, cells incubated with combination of 1 nM α-ZEL, 1 nM ZEN and 10 nM XAN appeared larger and had bigger nuclei (Figure 9), which might indicate a lower cell density. Similar results that could be observed for the other test compounds are shown in detail in the supplementary data (Figures S1, S2, S3).

## Discussion and conclusion

Evaluating not only the effects of a single compound, but its interplay with other bioactive substances is of growing relevance in toxicology and risk assessment, as the combinatory behavior of compounds can differ greatly from isolated effects. In particular, xenoestrogens seem to possess a high potential for interactions (8, 9). For example, our group recently showed the soy flavonoid genistein to potentiate estrogenic effects of ZEN in Ishikawa cells (9), thus raising the question on potential interactions of ZEN with other food-borne phytoestrogens. Considering beer as a potential source for ZEN intake, it was likely to expect interactions with flavonoids from hops, in particular with 8-PN, the most potent food-derived phytoestrogen known today.

Unexpectedly, the present *in vitro* data indicate that hop flavonoids might aid in protecting from adverse estrogenic effects of *Fusarium* toxins by antagonizing their ability to activate estrogen receptors. Especially XAN seems promising in that regard, as it significantly reduced the ER-dependent induction of AlP expression by both ZEN and α-ZEL in binary (Figure 4) and ternary (Figure 6) combinations, which also manifested in highly antagonistic CI values in the latter. In particular, the CI_50_ of 8.44 for the mixture “b” (ZEN:α-ZEL:XAN = 1:1:10) indicates a strong attenuating potency of XAN toward the estrogenicity of the two selected mycotoxins. The antagonistic behavior of mixture “b” also manifested at the level of cell growth induction and subsequent CI analysis (Figure 8), and seems also to be visible at the level of cell morphology (Figure 9), which further underlines the validity of these results. The strong antagonism observed in these ternary combinations (XAN/ZEN/α-ZEL) was connected to the presence of XAN, as the two *Fusarium* toxins interacted only marginally with each other (Figure 3). Even if labeled “moderately antagonistic” according to Chou (25) (Table 1) the combinatory effect of ZEN and α-ZEL should probably not be overinterpreted considering the quite high inherent deviations of the assay which cannot be taken into account by the CI analysis.

Interestingly, not only XAN, also 8-PN—despite acting as an ER agonist itself—interacted antagonistically with the selected mycotoxins in the AlP assay with CI_50_ values in the “antagonistic” range (1.45<CI<3), which stayed almost constant regardless of the applied concentrations (Figure 5). Adding 8-PN to the highly antagonistic mixture “b” and thus producing a quaternary combination of all four test compounds did not result in a further enhanced interaction, but rather in an attenuation of the antagonistic effect (Figure 7). Even as the antagonistic potential was clearly observed, it has to be concluded that XAN, and not 8-PN, is the main responsible agent for that interaction. We can also exclude that any of the observed antagonistic combinatory effects were caused by cytotoxicity, as SRB assays did not show any decrease of cell numbers over 2 d (data not shown) or 6 d of incubation (Figure 8).

XAN was already described as an anti-estrogenic compound previously, but the mechanism of that action is still not entirely identified. Gerhäuser proposed the inhibitory potential of XAN toward aromatase (CYP19) might reduce endogenous E2 levels (26), which would offer an explanation for its effects *in vivo*. Anyhow, there is increasing evidence for a more direct interaction with ER signaling. Unlike e.g., genistein, which is described as a tyrosine kinase inhibitor (27), XAN was reported to inhibit the phosphorylation of different serine kinases like Akt (28), protein kinase c and PDK1 (29). This could play a role in the observed antagonistic potential, as ER activation includes the phosphorylation of different serine residues of the receptor, in particular serine 167 (30), which could theoretically be inhibited by XAN. Another possibility was recently introduced by Yoshimaru et al. who found XAN to impair the functionality of the BIG3-PHB2 complex, which is involved in the modulation of ER signaling in MCF-7 cells (31). Thus, further studies are needed to elucidate the underlying mechanism of interaction.

Anyhow, our data not only reconfirm the anti-estrogenic activity of XAN, but also indicate that 8-PN, despite its innate estrogenic activity, shares the anti-estrogenic properties of its parent compound in combination with other estrogens at least to some extent.

Furthermore, we showed that XANs antagonistic potential is not limited to E2, but includes xenoestrogens like ZEN and α-ZEL, which might decrease their adverse effects on animals and humans. Further studies addressing the situation *in vivo* are required to explore this possibility.

Of note, the amounts of hop polyphenols needed to exert their antagonistic behavior toward ZEN and α-ZEL were in the low nanomolar range, concentrations that can realistically be expected to occur systemically after a moderate consumption of beer (32), thus underlining the relevance of these findings.

In summary, we hereby describe the two beer constituents XAN and (to a lesser extent) 8-PN to antagonize the isolated and combined estrogenic impact of *Fusarium* toxins in an *in vitro* test system, which seems of significance for risk assessment due to the possible simultaneous exposure to all those compounds, thus indicating the necessity for respective *in vivo* studies.

## Author contributions

GA was involved in designing the study, measured AlP and SRB assays, carried out statistical analysis, and wrote the manuscript. JB measured AlP assays with binary combinations containing 8-PN. DM was involved in designing and supervising the project and refined the manuscript.

### Conflict of interest statement

The authors declare that the research was conducted in the absence of any commercial or financial relationships that could be construed as a potential conflict of interest.

## Supplementary material

Figure S1 Impact on the proliferation of Ishikawa cells in the sulforhodamine B assay by the single compounds E2, ZEN, α-ZEL, 8-PN and XAN after 2x 72 h of incubation. Columns show the measured protein content of the wells in relation to the solvent control (0.15% (v/v) DMSO) as means + SD of at least 3 independent experiments. Significant differences to the respective no-effect dose were calculated by one-way ANOVA (*p* < 0.05), followed by Fisher's LSD *post-hoc* testing, and are indicated with “a” (E2), “b” (ZEN), “c” (?-ZEL) or “d” (8-PN). No significant difference was observed for XAN.

Figure S2 Bright field images of Ishikawa cells during a 2x 3d incubation with 0.2% (v/v) DMSO, 1 nM E2, 10 nM XAN, 1 nM ZEN, 1 nM α-ZEL or a combination of the latter three. Images were captured at day 0, 3 and 6 of the incubation at the same location of one well.

Figure S3 Images of Ishikawa cells after 3d and 6d of incubation with 0.2% (v/v) DMSO, 1 nM E2, 10 nM XAN, 1 nM ZEN, 1 nM α-ZEL or a combination of the latter three. After 3 and 6d of incubation, one well of each triplicate incubation was stained with Hoechst 33242 (nuclei, blue) and CellMask™ Deep Red Plasma Membrane Stain (cellular membranes, red) and the fluorescent image was captured. The figure shows both stainings separately and combined for each time-point.

## Abbreviations

8-PN: 8-prenylnaringenin
AlP: alkaline phosphatase
α-ZEL: α-zearalenol
CD-FCS: charcoal-dextran stripped fetal calf serum
DMSO: dimethyl sulfoxide
E2: 17β-estradiol
ER: estrogen receptor
ICI: ICI 182.780
LCIS: Live Cell Imaging Solution
PBS: phosphate buffered saline solution
SRB: sulforhodamine B
XAN: xanthohumol
ZEN: zearalenone
