# Supplementary figures and images for: The Hop Polyphenols Xanthohumol and 8-Prenyl-Naringenin Antagonize the Estrogenic Effects of Fusarium Mycotoxins in Human Endometrial Cancer Cells
Source: Front Nutr. 2018 Sep 19;5:85. doi: 10.3389/fnut.2018.00085 (PMC6156369; doi:10.3389/fnut.2018.00085)

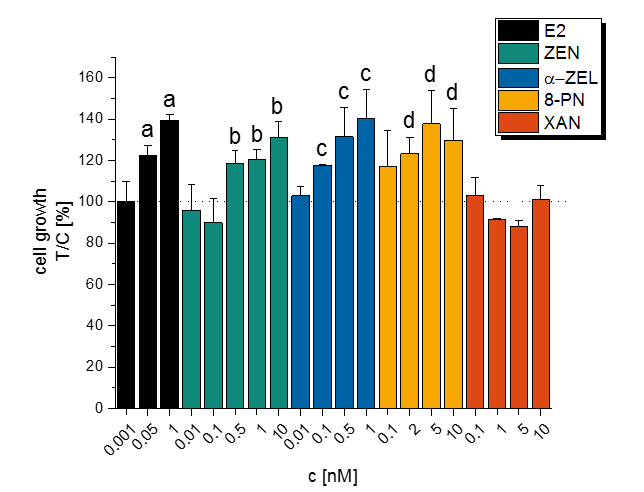

Supplement: Figure S1 — Impact on the proliferation of Ishikawa cells in the sulforhodamine B assay by the single compounds E2, ZEN, α-ZEL, 8-PN and XAN after 2x 72 h of incubation. Columns show the measured protein content of the wells in relation to the solvent control (0.15% (v/v) DMSO) as means + SD of at least 3 independent experiments. Significant differences to the respective no-effect dose were calculated by one-way ANOVA (p < 0.05), followed by Fisher's LSD post-hoc testing, and are indicated with “a” (E2), “b” (ZEN), “c” (?-ZEL) or “d” (8-PN). No significant difference was observed for XAN. [file Image_1.TIF]

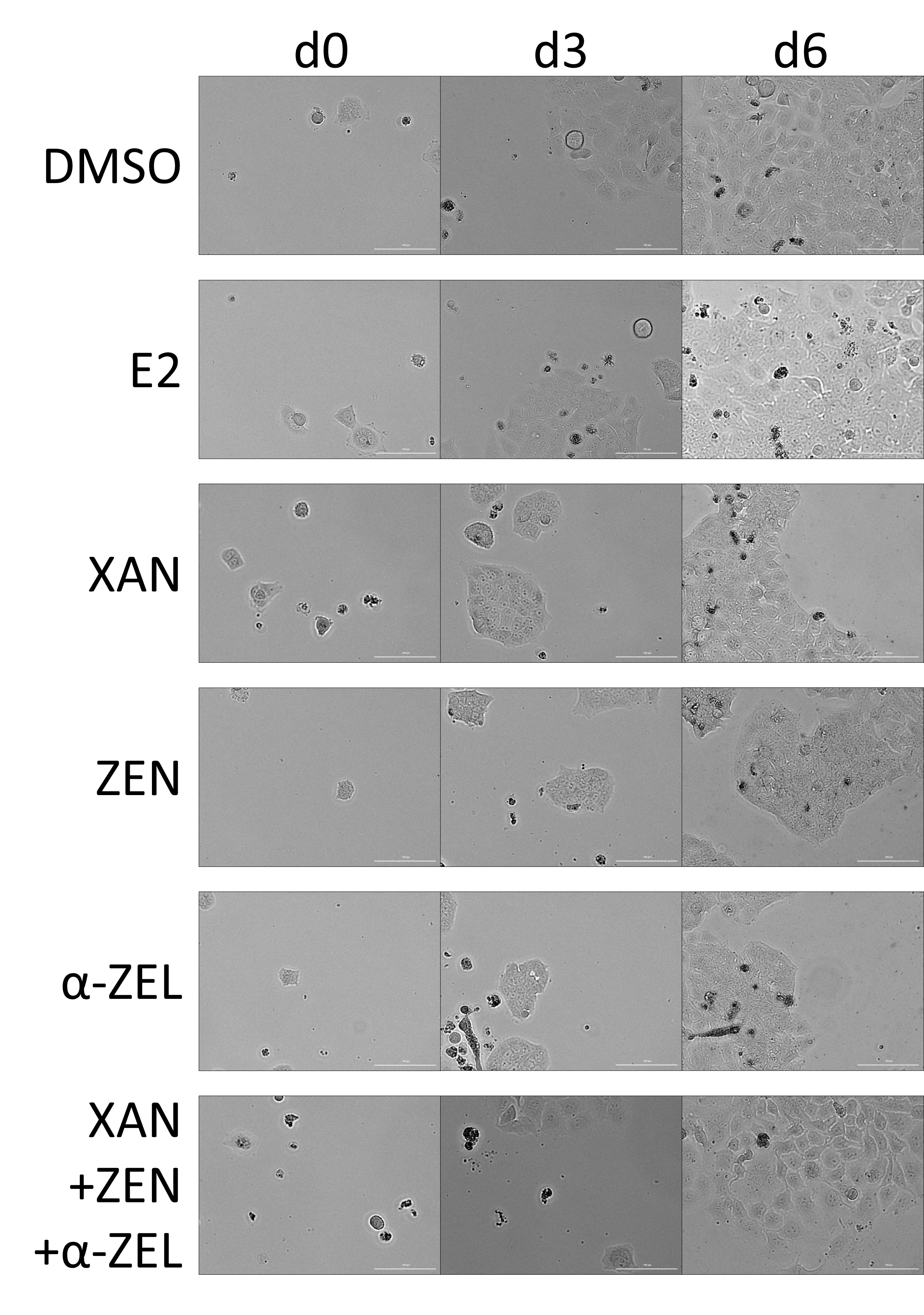

Supplement: Figure S2 — Bright field images of Ishikawa cells during a 2x 3d incubation with 0.2% (v/v) DMSO, 1 nM E2, 10 nM XAN, 1 nM ZEN, 1 nM α-ZEL or a combination of the latter three. Images were captured at day 0, 3 and 6 of the incubation at the same location of one well. [file Image_2.TIF]

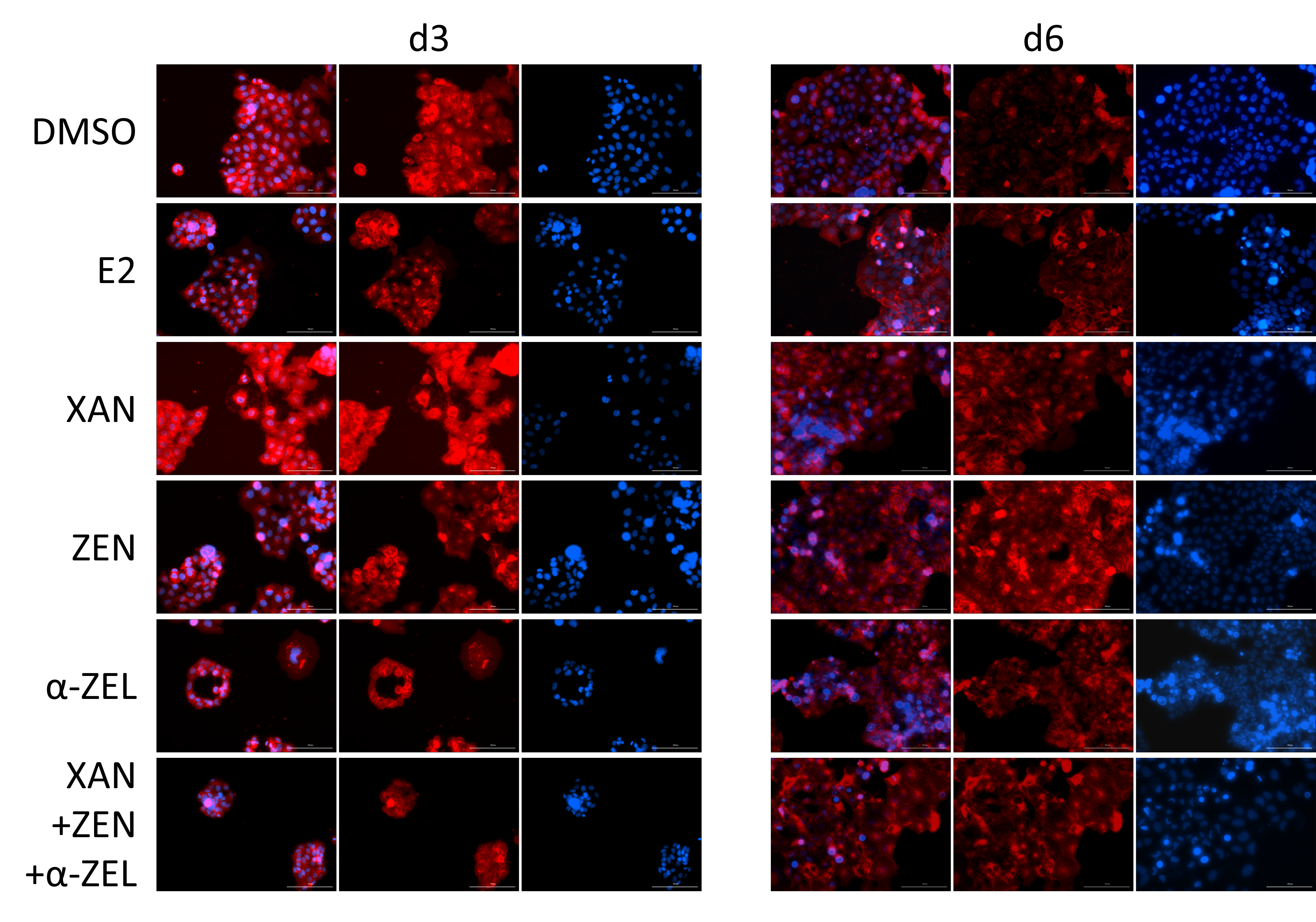

Supplement: Figure S3 — Images of Ishikawa cells after 3d and 6d of incubation with 0.2% (v/v) DMSO, 1 nM E2, 10 nM XAN, 1 nM ZEN, 1 nM α-ZEL or a combination of the latter three. After 3 and 6d of incubation, one well of each triplicate incubation was stained with Hoechst 33242 (nuclei, blue) and CellMask™ Deep Red Plasma Membrane Stain (cellular membranes, red) and the fluorescent image was captured. The figure shows both stainings separately and combined for each time-point. [file Image_3.TIF]
